# Feasibility and impact of an intensified antibiotic stewardship programme targeting cephalosporin and fluoroquinolone use in a tertiary care university medical center

**DOI:** 10.1186/1471-2334-14-201

**Published:** 2014-04-15

**Authors:** Johannes P Borde, Klaus Kaier, Michaela Steib-Bauert, Werner Vach, Annette Geibel-Zehender, Hansjörg Busch, Hartmut Bertz, Martin Hug, Katja de With, Winfried V Kern

**Affiliations:** 1 Department of Medicine, Abteilung Infektiologie, Universitätsklinikum Freiburg, Hugstetter Straße 55, D-79106 Freiburg i.Br, Germany; 2 Institute of Medical Biometry and Informatics, Freiburg i.Br, Germany; 3 Pharmacy Service, University Medical Center, D-79106 Freiburg i.Br, Germany; 4 Present address: Universitätsklinikum Carl Gustav Carus, Fetscherstraße 74, D-01307 Dresden, Germany

**Keywords:** Antibiotic stewardship, Interrupted time-series analysis, Cephalosporins, Fluoroquinolones

## Abstract

**Background:**

Restricted use of third-generation cephalosporins and fluoroquinolones has been linked to a reduced incidence of hospital-acquired infections with multidrug-resistant bacteria. We implemented an intensified antibiotic stewardship (ABS) programme in the medical service of a university hospital center aiming at a reduction by at least 30% in the use of these two drug classes.

**Methods:**

The ABS programme was focused on the 300-bed medical service. Prescription of third-generation cephalosporins was discouraged, whereas the use of penicillins was encouraged. Monthly drug use density was measured in WHO-ATC defined and locally recommended daily doses (DDD and RDD) per 100 patient days, to evaluate trends before (01/2008 to 10/2011) and after starting the intervention (1/2012 to 3/2013). The effect was analysed using interrupted time-series analysis with six non-intervention departments as controls.

**Results:**

Following initiation of the ABS intervention, overall antibiotic use in the medical service declined (p < 0.001). There was a significant intervention-related decrease in the use of cephalosporins and fluoroquinolones (p < 0.001) outperforming the decreasing baseline trend. Trend changes observed in some of the control departments were smaller, and the difference between trend changes in the medical service and those in control departments were highly significant for overall use and cephalosporin use reductions (p < 0.001) as well as for the increasing use of penicillins (p < 0.001). Mean use density levels (in RDD per 100 patient days) dropped for cephalosporins from 16.3 to 10.3 (−37%) and for fluoroquinolones from 17.7 to 10.1 (−43%), respectively. During the same period, the use of penicillins increased (15.4 to 18.2; 18%). The changes in expenditures for antibiotics in the medical service compared to control services minus programme costs indicated initial net cost savings likely to be associated with the programme.

**Conclusion:**

An intensified ABS programme targeting cephalosporin und fluoroquinolone use in the setting of a large academic hospital is feasible and effective. The intervention may serve as a model for other services and hospitals with a similar structure and baseline situation.

## Background

The steady increase in antimicrobial drug resistance is of growing concern. Strategies to reduce antimicrobial resistance essentially include limiting the inadequate use of antibiotics in both the hospital and primary care settings [1,2]. Earlier studies have shown that the reduced prescribing of aminoglycosides in hospitals may be associated with a reduction in the incidence of aminoglycoside-resistant gram-negative bacteria [3,4]. Recent observations show that reduced prescribing of particular other antibacterial drug classes, notably third-generation cephalosporins [5,6] and fluoroquinolones (FQ) [6,7], may reduce the incidence of hospital-acquired *C. difficile*[8-11], methicillin-resistant *Staphylococcus aureus* (MRSA) [12-14] and gram-negative bacteria producing extended-spectrum betalactamase (ESBL) [15,16].

Reducing the antibacterial drug use density in tertiary care centers may be challenging [17]. These centers serve as referral hospitals for patients with difficult to treat conditions and enhanced risk for complications including healthcare-associated infection that may require aggressive treatment. Often, such conditions include cancer, transplant or immunodeficiency or patients who are pretreated and being transferred because of treatment failure. However, practice guidelines commonly include treatment recommendations only for initial therapy. Given the limited evidence for second-line therapies, the often complex underlying disease and comorbidities of the tertiary care hospital patients, antimicrobial therapies in tertiary center patients often need to be individualized and based on expert consultation. Strategies to address inadequate therapy in such centers usually require an intensive infectious disease consultation service, frequent audits and feedback [1,18]. Whether relevant reductions in antimicrobial drug use density levels can be achieved here is uncertain. The tendency in many acute care hospitals is an increase in antimicrobial drug use density rather than a decrease [19,20] which in part is explained by decreasing lengths of stay and in part by an ever increasing number of patients with more complex diseases.

Freiburg University Hospital is a 1600-bed academic teaching hospital and tertiary care referral center with all major services and departments including renal, lung, heart and hematopoetic stem cell transplant centers. Locally consented infection management guidelines for the most frequent indications were first available in written form in 2006. In the following years, the overall antibiotic use density remained relatively stable. The use of fluoroquinolones increased slightly in the following years, in part due to the adoption of fluoroquinolone prophylaxis in neutropenia patients. The use of penicillins did not increase, the proportion of prescribed doses of penicillins within the betalactam class remained well below 50%, and ceftriaxone became one of the most prevalent antibacterial drugs. In 2010, we observed slightly increasing rates of enteric bacteria producing extended-spectrum betalactamases (ESBL) and vancomycin-resistant enterococci (VRE).

## Methods

### Setting and antibiotic stewardship programme

Freiburg University Hospital is a 1600-bed academic teaching hospital and tertiary care referral center with all major services and departments including renal, lung, heart and hematopoetic stem cell transplant centers. Locally consented infection management guidelines for the most frequent indications were first available in written form in 2006. In the following years, the overall antibiotic use density remained relatively stable. The use of fluoroquinolones increased slightly in the following years, in part due to the adoption of fluoroquinolone prophylaxis in neutropenia patients. The use of penicillins did not increase, the proportion of prescribed doses of penicillins within the betalactam class remained well below 50%, and ceftriaxone became one of the most prevalent antibacterial drugs. In 2010, we observed slightly increasing rates of enteric bacteria producing extended-spectrum betalactamase (ESBL) and vancomycin-resistant enterococci (VRE).

In 2011, we revised our internal guidelines and recommended penicillins as first-line drugs for many therapeutic indications while empirical cephalosporin and fluoroquinolone use were explicitly discouraged (“use more pens than cephs”, “don’t use combination therapy with FQs”, “abandon FQ prophylaxis in hematology-oncology”). The revised guidelines where consented, discussed in educational conferences and published in the intranet between July and October 2011. An intensified programme focusing on the 300-bed medical service was initiated to ensure compliance with the new guidelines and to achieve an at least 30% reduction in the prescribing of cephalosporins and fluoroquinolone within one year. This target of 30% was primarily derived from a benchmark analysis of tertiary care hospital antibiotic use data available through a surveillance and internal quality assurance programme (http://www.antiinfektiva-surveillance.de). The focus on the medical service was due to the facts that the medical service predominantly used third-generation cephalosporins and that >50% of all doses of third-generation cephalosporins and fluoroquinolones prescribed hospital-wide were in the medical service. We used the surgical service (including divisions of general surgery, thoracic, cardiac and vascular surgery, transplant surgery, and surgical critical care), urology, orthopedic surgery, neurology, neurosurgery, and the ENT department (all in total 750 beds) as controls. In the control departments no intensified ABS intervention took place.

The programme included four components: guideline revision, information and education, regular ward rounds and intensified infectious disease consultations, and feedback. Briefly, local clinical guidelines and pathways were revised by a multidisciplinary team including infectious disease physicians, clinicians from various divisions including hematology, emergency and intensive care medicine and general medicine, and clinical pharmacists. Guidelines discouraged the use of cephalosporins and fluoroquinolones, discouraged combination therapy, and encouraged the use of penicillins. The guidelines were made available in written pocket-like formats and were easily accessible through the hospital intranet. Educational events included initial short division-specific team briefings, summarizing the revised guidelines and explaining the overall strategy. We provided prospective audit with intervention and feedback in form of weekly rounds on “high antiinfective consumer wards” like the medical intensive care units and hematology units. The rounds focused on the appropriate choice of the drugs for empirical and targeted therapy, on dosing, and on early deescalation. In addition, quartely antibiotic use data per ward, service and division specifying trends in cephalosporin and fluoroquinolone versus broad-spectrum penicillin use were made available in the intranet to members of the infection management and control committee. Finally, the infectious disease service increased the number of bedside consultations in the medical service with academic detailing, individualized written recommendations, and follow-up consultations to enhance guideline adherence and compliance with the recommendations.

### Outcome data

Monthly antibiotic use data were obtained from the hospital pharmacy and were expressed as WHO-ATC defined daily doses (DDD) and dose definitions adapted to local guidelines to account for discrepancies between DDD for penicillins and commonly and locally recommended daily doses in hospital practice (recommended daily doses [RDD]) normalized per 100 patient (occupied bed) days. The data were administered using Microsoft® Access software and available on ward, division and service level and further aggregated as appropriate. Data on actual drug expenditures were also retrieved from the pharmacy database. No attempt was made to adjust for inflation. Programme cost data were estimated based on time sheets for personnel involved in the programme (senior physician, infectious disease fellow, pharmacist, data manager). The DRG case mix index, data on length of stay and on hospital deaths were obtained from the administration.

### Statistical analysis

The effect of the intervention on drug use density was analysed using interrupted time-series analysis for the periods between 1/2008 through 11/2011 as pre-intervention, and between 1/2012 through 3/2013 as post-intervention period. Basically, interrupted time-series analysis allows accounting for two effects of the intervention: a level-effect or a slope-effect. As our data indicate a change in the slope rather than a level change, our regression model to analyse the use of antibiotic *Y* over time *t* in a single setting was specified accordingly:

$$\begin{array}{ll} \;Y_{t}= & \beta_{0}+\beta_{1}\times \text{tim}e_{t}+\beta_{2}\times \text{time after interventio}n_{t}\\ & +u_{t} \end{array}$$

In detail, *β*_0_ determines the baseline level of the respective antibiotic use in January 2008, *β*_1_ determines the overall linear trend in the use of the respective antibiotic over the entire study period. *β*_2_ determines the change in post intervention trend where *time after intervention* is a continuous variable indicating the number of months after the start of the intervention and is coded as zero before the intervention. *u*_*t*_ represents the error term.

We applied the same interrupted time-series approach for the control departments and compared the post-intervention trend changes (*β*_2_) in the medical service with the average effect (*β*_2_) in the control departments. All analyses were carried out using Stata 12 (StataCorp, College Station, TX, USA). We applied linear regressions with Newey-West standard errors with a maximum lag of 2 to be considered in the autocorrelation structure. Stata’s lincom command was applied to subtract the average *β*_2_ in the non-intervention settings from the *β*_2_ in the medical service.

Descriptive statistical analysis was performed by using Microsoft Excel® Software. Heteroskedastic t-test was applied to compare mean drug use densities before and after the intervention. Mann–Whitney U test was used to compare drug expenditures before and after the programme.

## Results

**Figure 1:**
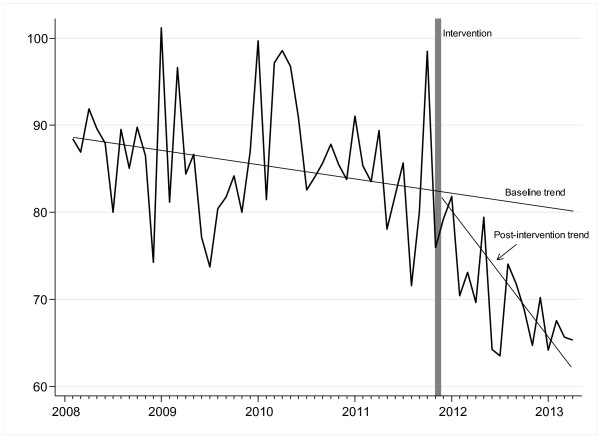
Trends in the overall monthly antibiotic use density (expressed as RDD per 100 patient days) in the medical service pre- and post-intervention.

Following the initiation of the intensified ABS programme, the overall antibiotic use density in the medical service declined by 14-20%. The mean use density values before and after the intervention were 110.5 versus 94.8 DDD per 100 patient days (−14.2%, P < 0.001) or 86.1 versus 68.8 RDD per 100 patient days (−19.9%, P < 0.001), respectively (Table 1 and Figure 1). This was explained by a decreasing use of both cephalosporins (in particular third generation cephalosporins) and fluoroquinolones. The mean use density levels for cephalosporins dropped from 16.3 to 10.3 RDD per 100 patient days (−36.8%, P < 0.001), and for fluoroquinolones the decline was from 17.7 to 10.1 RDD per 100 patient days (−43.2%, P < 0.001), respectively. These trends were similar when they were calculated in DDD per 100 patient days (−30%, and −39%, respectively) (Table 1). During the same period, the prescription of penicillins increased by 18% (RDD per 100 patient days) to 28% (DDD per 100 patient days) (Table 1). Monitoring hospital deaths and DRG case-mix index ensured that changes in the above outcomes were not related to a change in these variables (data not shown).

**Table 1:** Comparison of mean monthly drug use density values expressed as DDD per 100 patient days or RDD per 100 patient days in the medical service pre- and post-intervention

	**DDD/100**				**RDD/100**			
**Drug class**	**Pre-**	**Post-**	**Percent change**	**P value**	**Pre-**	**Post-**	**Percent change**	**P value**
Cephalosporins	20.1	14.0	−30.2%	<0.001	16.3	10.3	−36.8%	<0.001
3° Cephalosporins	14.4	7.4	−49.0%	<0.001	13.1	6.4	−51.3%	<0.001
1°/2° Cephalosporins	5.6	6.6	+17.7%	<0.05	3.2	3.9	+23.6%	<0.01
Penicillins	23.1	29.6	+28.3%	<0.001	15.4	18.2	+17.9%	<0.001
Piperacillin ± BLI	9.3	9.9	+5.8%	ns	10.9	11.5	+5.8%	ns
Aminopenicillins + BLI	6.6	11.3	+69.9%	<0.001	2.4	4.1	+70.4%	<0.001
Narrow-spectrum penicillins	7.1	8.5	+19.1%	ns	2.1	2.5	+20.3%	ns
Carbapenems	9.5	8.3	−12.7%	<0.01	6.9	5.8	−15.7%	<0.001
Fluoroquinolones	19.6	12.0	−38.5%	<0.001	17.7	10.1	−43.2%	<0.001
Aminoglycosides	0.9	0.7	−18.9%	ns	0.7	0.6	−19.1%	ns
Glycopeptides	4.3	3.9	−8.6%	ns	4.3	3.9	−8.6%	ns
Tetracyclines	1.4	0.9	−36.9%	<0.01	1.0	0.7	−35.9%	<0.01
Macrolides/clindamycin	18.1	13.7	−24.4%	<0.001	10.3	7.9	−22.8%	0.001
TOTAL	110.5	94.8	−14.2%	<0.001	86.1	68.9	−19.9%	<0.001

Beta-lactamase inhibitor (BLI).

**Figure 2:**
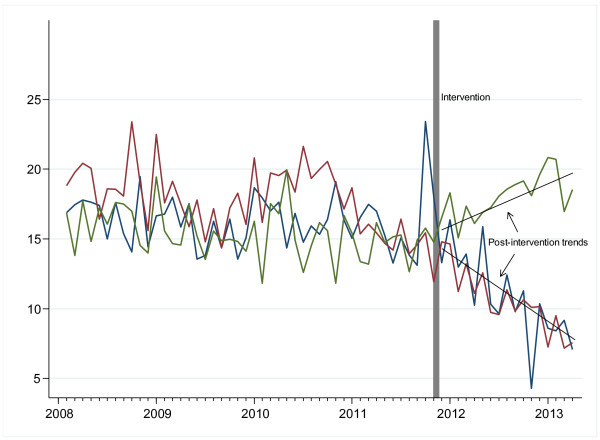
**Trends in the monthly antibiotic use density (expressed as RDD per 100 patient days) for cephalosporins (blue).** Fluoroquinolones (red) and penicillins (green) in the medical service pre- and post-intervention.

We next analysed trend changes using monthly drug use levels expressed in RDD per 100 patient days in an interrupted time-series model. Using the single-level model, it was shown that the use of cephalosporins already declined slightly before the intervention (0.0169 RDD per 100 patient days each month), but an additional major decrease in cephalosporin use (0.517 RDD per 100 patient days each month, P < 0.001) was seen after the intervention (Table 2 and Figure 2). The trend changes for fluoroquinolone use post-intervention were similar and also statistically significant (Table 2 and Figure 2). The model indicated that penicillin use increased substantially and significantly after the intervention (0.34 RDD per 100 patient days per month). Separate analyses of the data for the medical ICU, hematology-oncology and other internal medicine wards indicated that the reduced fluoroquinolone prescription was primarily related to trend changes in hematology-oncology, and the reduced cephalosporin prescription post-intervention was particularly strong in the medical ICU (Table 3).

**Table 2:** Monthly drug use density (expressed as RDD per 100 patient days) trends in the medical service pre- (baseline) and post-intervention as estimated in a single-level interrupted time-series model (P values in parentheses)

	**Cephalosporins**	**Fluoroquinolones**	**Penicillins**	**TOTAL**
Baseline	−0.02	−0.10	−0.03	−0.13
trend β_1_	(ns)	(<0.001)	(ns)	(0.038)
Post-intervention	−0.52 (<0.001)	−0.40 (<0.001)	+0.34 (<0.001)	−1.10 (<0.001)
trend change β_2_				
Intercept β_0_	16.61 (<0.001)	19.96 (<0.001)	16.11 (<0.001)	88.73 (<0.001)
N	63	63	63	63

**Table 3:** Monthly drug use density (expressed as RDD per 100 patient days) trends in hematology-oncology (Hem-Onc) and the medical ICU pre- (baseline) and post-intervention as estimated in a single-level interrupted time-series model; only trend changes that were statistically significant are shown

		**Cephalosporins**	**Fluorochinolones**	**Penicillins**	**TOTAL**
Baseline trend β_1_	Hem-Onc	−0.05 (ns)	−0.18 (<0.001)	−0.00 (ns)	−0.17 (ns)
	Medical ICU	−0.045 (ns)	−0.02 (ns)	−0.49 (<0.001)	−0.31 (ns)
Post-intervention	Hem-Onc	−0.41 (<0.001)	−1.16 (<0.001)	+0.17 (ns)	−2.17 (<0.001)
trend change β_2_	Medical ICU	−1.06 (<0.05)	+0.04 (ns)	+1.83 (<0.001)	−1.11 (ns)
Intercept β_0_	Hem-Onc	14.06 (<0.001)	38.29 (<0.001)	18.88 (<0.001)	124.1 (<0.001)
	Medical ICU	26.87 (<0.001)	13.02 (<0.001)	40.93 (<0.001)	148.3 (<0.001)
N		63	63	63	63

The multi-level model confirmed the effect of the intervention on the medical service compared with the control departments (Table 4). Interestingly, there was also some trend change in some control departments around the time of the intervention, but it did not reach the magnitude of the trend change seen in the medical service. The reduction of cephalosporins (p < 0.001) and overall antibiotic use (p < 0.001) as well as the increase in penicillin prescriptions (p < 0.001) following the intervention remained significant when compared with the control departments.

**Table 4:** **Monthly drug use density (expressed as RDD per 100 patient days) trends ß**_**2 **_**in the medical service post-intervention compared to the effects of a hypothetical intervention at six control services according to regression analysis (P values in parentheses)**

	**Post-intervention trend changes β**_**2**_			
**Service/department**	**Cephalosporins**	**Fluoroquinolones**	**Penicillins**	**TOTAL**
Medical service	−0.517	−0.40	0.34	−1.102
(intervention)	(<0.001)	(<0.001)	(<0.001)	(<0.001)
Department A	−0.171	0.01	0.261	0.247
(control)	(0.024)	(ns)	(<0.001)	(0.041)
Department B	−0.40	0.451	−0.0579	0.0246
(control)	(<0.001)	(<0.001)	(0.1)	(ns)
Department C	0.057	−0.315	0.0501	−0.332
(control)	(ns)	(<0.001)	(ns)	(0.006)
Department D	−0.134	−0.0431	0.0418	−0.272
(control)	(0.077)	(ns)	(ns)	(0.024)
Department E	−0.278	−0.021	−0.0305	−0.534
(control)	(<0.001)	(ns)	(ns)	(<0.001)
Department F	−0.426	0.002	0.206	−0.454
(control)	(<0.001)	(ns)	(<0.001)	(<0.001)
Medical service	−0.292	−0.414	0.261	−0.882
versus controls*	(<0.001)	(<0.001)	(<0.001)	(<0.001)

*Stata’s lincom command was applied to simply subtract the average β_2_ in the control (non-intervention) settings (rows 2–7) from the β_2_ in the medical service in which the intervention took place.

As a secondary objective we evaluated potential net savings associated with the programme (drug expenditures changes compared to control services minus programme costs) in a simple before/after analysis. The mean annual drug expenditures on all antibiotics in the medical service pre-intervention were 1,038,648 € decreasing to 594,684 € in the post-intervention period (−43%) while in the control departments there was also a downward trend which, however, was much smaller (−24%, 781,440 € pre-intervention, 595,956 € post-intervention), giving a conservative estimate of at least 248,000 € annual cost reduction (−19% instead of −43%) in the medical service. The team involved in the intensified medical service-focussed programme was a senior physician with a dedicated effort to the programme of 35%, an infectious disease fellow (75%), a pharmacist (12%), and a data manager (6%) corresponding to yearly personnel costs of 102,950 €. Thus, estimated initial annual net savings of at least 145,000 € were likely to be associated with the programme.

## Discussion

Interrupted time-series (ITS) analysis is regarded as the statistic tool of choice to differentiate between baseline trends and true intervention-related effects of ABS programmes. In the recently published Cochrane metaanalysis [18] most of the 86 finally included studies with adequate quality were designed as ITS analysis, underscoring the importance of this type of evaluation in field of ABS [18,21]. In fact, we considered it as essential to measure and evaluate time trends for antibiotic use, both in and outside the primary target service by ITS analysis to estimate the contribution of our additional efforts optimizing antimicrobial drug treatment. Of note, a trend of fewer fluoroquinolone prescriptions was already observed prior to the programme initiation, and was related to a decision taken earlier in 2011 to abandon fluoroquinolone prophylaxis in hematology-oncology patients [22].

Epidemiological data from Germany [19,23] indicate a dominance in acute-care hospitals of cephalosporins over penicillins, and a frequent use of fluoroquinolones. We were surprised to see that despite considerable efforts, cephalosporins had remained the dominant antibacterial drug class in this hospital, at least if RDD rather than DDD were counted. There is a well-established association between the use of third generation cephalosporins, fluoroquinolones and the emergence and spread of multidrug-resistant bacteria and *Clostridium difficile* at hospital level [8,22,24,25]. Moreover, it has been shown that a substantial part of the MRSA related costs could be prevented if the use of fluoroquinolones and cephalosporins was reduced [5]. Previous studies have demonstrated significant decreases in cephalosporin and fluoroquinolone use with various outcomes [6,10,26,27]. Some studies have not assessed overall drug or drug-class consumption, other reported a compensatory increase in carbapenem prescriptions. There still remains the question of feasibility and sustainability of programmes in particular in large acute-care hospitals without inducing negative compensatory side-effects. Tertiary referral hospitals are often challenging sites for antibiotic stewardship interventions given the complex patient conditions and the involvement of various medical specialties with different department structures and internal guidelines. The present programme essentially addressed and implemented a revised policy of recommending penicillins as first-line drugs for many therapeutic indications. We encouraged the preferential use of penicillin, flucloxacillin, and ampicillin-sulbactam rather than cephalosporins (and/or fluoroquinolones) for a number of indications. Our results demonstrate that it is feasible to reduce cephalosporin and fluoroquinolone use by >30% within 12 months in the setting of a large tertiary university hospital using a relatively simple bundle approach. As intended, we observed a significant increase in penicillin prescriptions whereas the overall antibiotic use showed a decline which appeared to be attributed to the programme and is a favourable “side effect”. The use of carbapenems dropped, despite we report a slightly increasing rate of ESBL isolates since 2010. This may reflect a more rational use of this drug class after the intervention, and indicates an overuse in the pre-intervention period. Similar effects were observed in the class of tetracyclines and macrolides.

The sustainability of the programme and its effects need to be determined, and the efficacy of a similar policy in other services of our hospital needs to be demonstrated. Outcomes on resistant bacteria were not assessed in this study. Preliminary analysis shows an impact on the incidence of infection due to *C. difficile* and the frequency of vancomycin-resistant enterococci. A formal analysis using ITS and a more appropriate modeling of possibly delayed effects on nosocomial (versus community-acquired) infection will be needed to fully understand the impact of the programme on resistant bacteria. We are particularly interested to measure effects over time on third-generation cephalosporin-resistant enteric bacteria since there is a lack of data allowing to assess the role if any of hospital-based interventions on the epidemiology of this group of microorganisms.

The investment needed for the programme was moderate. The priorities of the existing infectious disease consultant service were redefined, and, with some additional personnel resources, the achievement of net cost savings was very likely. Our estimate of net savings of almost 150,000 € for a 300-bed service (i.e. ~500 € per bed) is an important finding but was derived from a simple before/after analysis that cannot differentiate between truly intervention-related savings and other effects. No attempt was made to quantify other potential cost savings such as shorter lengths of stay with better utilization of the inpatient service, impact on antiviral and antifungal drug use, or fewer infectious complications due to resistant bacteria and *C. difficile*. Interestingly, recent experience from North America indicate very similar initial net cost savings associated with an ABS programme if only antibacterial drugs were assessed, and much higher cost savings when antifungal drugs were included in the analysis [17].

## Conclusion

In conclusion, an intensified ABS programme targeting cephalosporin und fluoroquinolone use in the setting of a large academic hospital is feasible and effective. Our intervention may serve as a model for transferring this or similar programs to other services and to other hospitals with similar structures and a similar baseline situation.

### Ethics statement

The revised guidelines where consented, discussed in educational conferences and published in the intranet between July and October 2011. All departments of the University Medical Center and the hospital administration agreed on the internal guidelines, ABS interventions and publication of epidemiological data. The ethics committee was notified about the trial - formal approval was not required, because the project is based on epidemiological data. Research involving human subjects, human material, specific human or personalized data was not carried out.

## Competing interests

All authors declare no competing interests. Funding: This study was supported in part by internal funds from the Department of Medicine. WVK and KdW were supported in part by a grant from the Federal Ministry of Health (BMG grant IIA5-2011-2511FSB340).

## Authors’ contributions

JPB wrote the manuscript, he was involved in the ID consultant service during the intervention period and performed descriptive statistical analysis of the data. KK did all advanced statistical work and critically reviewed the manuscript. MS-B designed and adopted the database system for ABS purposes, WV supported us regarding advanced statistical questions, AG-Z implemented consented ABS guidelines in the intensive care units, HanB and HarB implemented and discussed internal guidelines for the emergency medicine department and hematology/oncology. MH provided prescription and consumption data from the pharmacy service. WVK and KdW were planning the project and designed the ABS interventions. WVK supervised the clinical ID consultant service. All authors read and approved the final manuscript.

## Pre-publication history

The pre-publication history for this paper can be accessed here:

http://www.biomedcentral.com/1471-2334/14/201/prepub
